# A qualitative study on parents’ illness perceptions and psychosomatic experiences of adolescent patients with emotional disorders caused by school bullying

**DOI:** 10.3389/fpsyg.2026.1682557

**Published:** 2026-03-02

**Authors:** Xiaofang Lin, Lili Pan, Lamei Yu, Cheng Hang, Yinghui Li

**Affiliations:** Huai'an No.3 People's Hospital, Huai'an, Jiangsu, China

**Keywords:** adolescents, cognition, emotional disorders, parents, psychosomatic experience, qualitative research, school bullying

## Abstract

**Objective:**

To gain an in-depth understanding of the cognition and psychosomatic experiences of parents whose adolescent children have developed emotional disorders due to school bullying.

**Methods:**

This qualitative study employed purposive sampling to select 14 parents of adolescent patients with emotional disorders caused by school bullying, who were treated at Huai’an Mental Health Center from August 2023 to October 2024. Semi-structured interviews were conducted, and the interview data were analyzed using Colaizzi’s seven-step analysis method.

**Results:**

Four themes were extracted regarding parents’ cognition and psychosomatic experiences: insufficient understanding of the disease, various negative psychological experiences, decline in social functioning and quality of life, and a strong desire for support and help from all sectors of society.

**Conclusion:**

Parents generally lack knowledge about emotional disorders resulting from school bullying and have cognitive misunderstandings. They experience severe negative psychosomatic symptoms. Medical staff should pay close attention to parents’ cognitive status and their adverse psychosomatic experiences, helping them restore good social functioning and quality of life. Establishing a comprehensive management and support system for adolescent patients with emotional disorders caused by school bullying can promote early recovery from psychological distress, restore mental peace, and facilitate a full return to their roles.

## 1 Introduction

Bullying is defined as intentional, repetitive aggressive behavior grounded in a power imbalance (Olweus, 2013). The Norwegian psychologist Olweus, D. defined victims and bullies as early as 1993. Victims of bullying are individuals who are repeatedly subjected to deliberate harm within an unequal power relationship, and who consequently experience a range of physical and psychological suffering and negative outcomes. Bullies refer to individuals—or dominant members of a group—who, within power-imbalanced relationships, intentionally and repeatedly engage in aggressive behaviors to cause physical or psychological distress to others (Olweus, 1993). Both inside and outside school settings are among the most common contexts in which bullying occurs.

In 2016, the State Council of the People’s Republic of China issued the Notice on the Implementation of Special Governance of School Bullying, which defined school bullying as intentional or malicious acts carried out by students against other students, both inside and outside school, through physical, verbal, or online means, resulting in physical injury, property loss, or psychological harm to the victim. According to the methods employed, bullying can be classified into physical bullying, verbal bullying, cyberbullying, and relational bullying (The State Council General Office of Education Supervision Committee, 2016). Among these, cyberbullying is a product of the rapid development of digital technologies in recent years and represents a new form of school bullying, constituting one of the negative consequences of online social interaction (Lee et al., 2017).

These complex bullying experiences pose serious threats to victims’ psychological well-being, social functioning, and academic development, and represent a major external stressor contributing to the development of clinical emotional disorders in some adolescents (Arseneault et al., 2010). Emotional disorders comprise a group of psychological conditions primarily characterized by anxiety, depression, fear, and obsessive symptoms. Due to the combined influence of age, genetic vulnerability, and environmental factors—and because their physical and psychological development is not yet mature—adolescents constitute a major high-risk population for emotional disorders (Wu et al., 2022).

According to a report by the United Nations Educational, Scientific and Cultural Organization, bullying by peers globally, almost one in three students has been bullied in the past month (United Nations Educational, Scientific and Cultural Organization, 2019). In China, the reported prevalence of bullying victimization and perpetration were 66% and 37% (Wu et al., 2022; Xiao et al., 2022). A substantial body of research has confirmed that bullying significantly increases the risk of emotional disorders in adolescents, and that the severity of psychological problems is positively correlated with the degree of bullying exposure (Zhao et al., 2024). For example, a meta-analysis by Moore et al. (2017) found that victims of bullying had 2.77 times higher risk of depression and 2.39 times higher risk of anxiety compared with non-victimized peers. These findings indicate a clear and severe psychopathological association between bullying and emotional disorders.

In recent years, increasing research attention has been devoted to adolescent bullying. For instance, Fraguas et al. (2021) conducted a high-quality meta-analysis of school-based anti-bullying intervention programs, demonstrating that systematic, school-level interventions can effectively reduce bullying incidence and improve students’ mental health outcomes, thereby providing high-level evidence for practice. Meanwhile, with the advancement of digital technologies, growing attention has been paid to the additive effects of cyberbullying and traditional bullying. A large-scale study by Chudal et al. (2022) across 13 European and Asian countries found that adolescents exposed to both traditional bullying and cyberbullying experienced significantly higher levels of psychological distress than those exposed to only one form, highlighting the substantial harm of combined bullying victimization. Some studies have further indicated that individuals who experienced peer bullying in childhood face risks of anxiety, depression, and suicidal ideation in adulthood (up to 45 years of age) that are comparable to—or even greater than—those associated with childhood maltreatment by adults (Lereya et al., 2015).

To gain a deeper understanding of the impact of school bullying on adolescents and their families, bullying must be examined within a multilayered, interactive ecological system. Drawing on Bronfenbrenner’s ecological systems theory, this study conceptualizes bullying as a traumatic event that primarily occurs within the microsystem (e.g., schools and peer groups), while recognizing that its impact transcends system boundaries, directly affecting the adolescent’s core microsystem—the family. The family is not a passive recipient of stress but a dynamic, adaptive holistic system (Family Systems Theory). This implies that significant stress experienced by any family member—such as an adolescent developing an emotional disorder as a result of bullying—will disrupt the equilibrium of the entire system, eliciting cognitive, emotional, and behavioral responses from all members, particularly parents. Conversely, family responses constitute the most proximal and influential environment shaping the patient’s recovery process.

From this perspective, the family plays a dual core role in bullying-related crises. First, the family functions as the primary “stress-bearing system.” When a child experiences bullying and develops an emotional disorder, parents often undergo a family crisis. In addition to managing their own feelings of shock, anger, self-blame, and helplessness, parents may face multiple stressors, including caregiving burden, financial pressure, social stigma, and difficulties communicating with schools or healthcare systems, which may severely compromise their physical and mental health as well as social functioning. Second, the family also serves as an indispensable “key resource system” in the adolescent’s recovery. Parents’ beliefs, emotions, and behaviors directly shape the emotional climate and support environment of the patient. A well-functioning family can provide a secure base that facilitates recovery from trauma, whereas a family that is already vulnerable or becomes dysfunctional during the crisis may unintentionally intensify the child’s isolation and psychological distress.

As the core microsystem of adolescent development, the role of the family in bullying incidents has attracted increasing scholarly attention. A longitudinal study by Bowes et al. demonstrated that children from low-cohesion, high-hostility family environments were at significantly higher risk of developing internalizing problems, such as emotional disorders, following bullying victimization (Bowes et al., 2010). Moreover, parents’ perceptions of bullying and their coping responses directly influence the course of events. When parents misinterpret bullying as “playful interactions between children” or adopt avoidant coping strategies, opportunities for timely intervention may be missed, thereby exacerbating adolescents’ feelings of helplessness and social isolation (Ostrov et al., 2022).

In contrast, domestic research in this area remains at an early stage. Through interactions with psychiatrists, psychologists, nurses, and adolescents suffering from emotional disorders caused by school bullying, as well as their primary family members, it has been observed that family members’ misunderstandings of the illness and their psychological and physiological reactions upon learning of the child’s diagnosis pose significant challenges for both families and clinicians. In severe cases, these issues may even interfere with treatment completion, highlighting a clear clinical need for research in this area. Importantly, the present study adopts a parent-centered perspective, which has been largely absent in domestic research to date, and thus seeks to fill this critical research gap.

Accordingly, this study does not conceptualize the family merely as a provider of support, but rather as a “co-experiencer” and “secondary victim” of bullying-related crises, thereby offering a more comprehensive understanding of the overall stress burden borne by the family system. Based on this framework, the present study proposes the following progressive research questions to systematically elucidate parents’ full experiences during this crisis:

(1)How do parents perceive the illness-related association between bullying and emotional disorders in their children?
(2)How do these perceptions influence parents’ emotional and behavioral responses?
(3)How do these cognitions and caregiving pressures manifest as psychological distress and psychosomatic symptoms in parents?
(4)What coping resources and strategies do parents adopt in the face of dual individual and family pressures, and what difficulties do they encounter?
(5)Based on their lived experiences, what core needs and recommendations do parents propose for the development of an effective external support system?

Based on these questions, this study conducts in-depth interviews with parents of adolescents who developed emotional disorders as a result of school bullying, with the aim of providing empirical evidence to inform the development of targeted prevention and intervention strategies.

## 2 Subjects and methods

### 2.1 Research subjects

A purposive sampling method was employed to recruit parents of adolescents with emotional disorders caused by school bullying who were treated at the Huai’an Mental Health Center between August 2023 and October 2024. The inclusion criteria were as follows:

(1)The adolescent’s diagnosis met the relevant criteria of the Chinese Classification and Diagnostic Criteria of Mental Disorders (CCMD-3) and was confirmed as an emotional disorder by two attending psychiatrists or physicians with senior professional titles;
(2)A disease duration of less than 1 year;
(3)The adolescent was aged 11–18 years (Su, 2014);
(4)The adolescent’s emotional disorder was triggered by school bullying;
(5)The parent had an educational level of primary school or above and demonstrated adequate communication ability;
(6)Voluntary participation in the study with written informed consent provided.

The exclusion criteria were:

(1)Presence of severe physical illness;
(2)Parents who, following evaluation by a psychiatrist, were found to have significant psychiatric disorders (e.g., major depressive episodes, schizophrenia) or cognitive impairments that might affect their ability to understand the interview content or express themselves coherently.

Sample size determination was guided by the principle of information saturation, defined as the point at which no new themes emerged. A total of 14 participants were ultimately included and assigned identification codes N1–N14. Their general demographic characteristics are presented in Table 1.

**Table 1:** General information of interviewees (*n* = 14).

Interviewee	Gender	Education level	Age	Occupation	Marital status	Place of residence	Monthly income
N1	Female	Junior High	40	Farmer	Married	Jiangsu Province	3,000-5000yuan
N2	Female	Junior High	38	Worker	Married	Jiangsu Province	5,000-8000yuan
N3	Female	Elementary	55	Farmer	Married	Jiangsu Province	3,000-5000yuan
N4	Male	High School	40	Technician	Married	Jiangsu Province	6,000-9000yuan
N5	Female	Junior High	45	Worker	Married	Shandong Province	5,000-8000yuan
N6	Male	High School	39	Worker	Remarried	Jiangsu Province	5,000-8000yuan
N7	Female	Junior High	49	Farmer	Married	Jiangsu Province	3,000-5000yuan
N8	Male	High School	45	Farmer	Widowed	Jiangsu Province	3,000-5000yuan
N9	Male	College	40	Clerical Staff	Married	Anhui Province	6,000-9000yuan
N10	Male	Junior High	44	Worker	Spouse Missing	Anhui Province	5,000-8000yuan
N11	Female	Junior High	42	Unemployed	Married	Jiangsu Province	3,000-5000yuan
N12	Female	Junior High	38	Unemployed	Married	Jiangsu Province	3,000-5000yuan
N13	Male	Junior High	52	Farmer	Married	Jiangsu Province	3,000-5000yuan
N14	Female	Junior High	53	Farmer	Married	Jiangsu Province	3,000-5000yuan

This study was approved by the Ethics Committee of Huai’an Third People’s Hospital. (Research)−2021–02.

### 2.2 Methods

#### 2.2.1 Research methodology

A phenomenological approach in qualitative research was adopted. We conducted one-on-one, face-to-face, in-depth semi-structured interviews with the 14 interviewees. Based on the research purpose and literature review, and after discussions among the research team members, an interview outline was developed. The interview consisted of a series of open-ended questions, as follows:

1. When did you find out that your child was bullied at school? How did you handle it?
2. Please specifically talk about your psychosomatic experiences and feelings after knowing your child was bullied and even became ill.
3. How much do you know about “adolescent emotional disorder” caused by school bullying?
4. What methods have you used to help improve your own psychosomatic state? How effective were they?
5. What methods have you used to help stabilize your child’s emotions? How effective were they?
6. What help have you received after your child developed an emotional disorder? What kind of help do you most desire now? In what form?

As the interview was drawing to a close, we asked, “Is there anything else you would like to add about this interview?” to gather additional information.

#### 2.2.2 Data collection

One week prior to the formal interviews, the researcher contacted participants via WeChat or QQ and distributed the interview guide through these platforms. The interview time and location were scheduled 1–2 days in advance. All interviews were conducted in a quiet psychological counseling room within the inpatient ward, with soft lighting and minimal disturbance.

All interviews were conducted by one researcher, with only one participant interviewed per day. Prior to each interview, the researcher explained the purpose, content, and significance of the interview, emphasized strict adherence to confidentiality principles, and used coded identifiers instead of participants’ names to protect privacy. Each interview lasted approximately 30–40 min, and each participant was interviewed once or twice. Interviews were audio-recorded with participants’ consent, and the researcher simultaneously documented non-verbal behaviors, including eye contact, facial expressions, tone of voice, and speech rate.

Participants were encouraged to freely express their feelings and experiences, and the interviewer employed qualitative interviewing techniques such as open-ended questioning, probing, reflective questioning, and clarification as appropriate. When participants displayed negative emotions, the interviewer provided appropriate emotional reassurance but avoided leading or suggestive statements to minimize information bias.

Following each interview, the audio recordings were promptly transcribed verbatim, and the transcripts were organized in conjunction with interview notes. The processed data were then returned to participants for verification to enhance accuracy and credibility.

Sample size determination followed the principle of information saturation in qualitative research. Data collection and preliminary analysis were conducted concurrently to allow for a dynamic assessment of saturation. Specifically, after approximately 10 interviews, the research team initiated preliminary coding and theme development based on the collected transcripts, which served as a reference framework for subsequent interviews. Thereafter, with the addition of each 1–2 new interviews, newly collected data were immediately compared with the existing analytical framework to determine whether any new codes, concepts, or themes relevant to the core research questions emerged.

When analysis of three consecutive newly added interviews yielded no new information that could substantially extend or modify the existing thematic framework, data saturation was considered to have been achieved. Throughout this process, the research team engaged in regular discussions to maintain a cautious and shared consensus regarding the definition of “new information.” Based on this assessment, information saturation was confirmed after the 12th interview.

To enhance the richness of the sample and the thickness of the descriptions, and in consideration of ethical responsibility toward participants who had already consented to participate, all 14 planned interviews were ultimately completed. All interview data were included in the final analysis to ensure the completeness and robustness of the study’s conclusions.

#### 2.2.3 Data analysis

Within 24 h after each interview, the interview data were imported into ATLAS.TI 9.0 software for data management and analysis. While retaining the completeness of individual viewpoints and statements, the psychological and physical experiences of the interviewees were analyzed and explored to enhance the credibility of the analysis. Colaizzi’s seven-step analysis method (Liu, 2019) was used:

1. Read all interview data carefully.
2. Extract significant statements.
3. Encode repetitive and meaningful viewpoints.
4. Cluster the encoded viewpoints.
5. Write detailed descriptions.
6. Summarize similar viewpoints.
7. Return to the interviewees for verification.

#### 2.2.4 Quality control

1. By reviewing the literature, an initial interview outline was developed, which was then optimized based on reviews by two chief physicians in child psychiatry, one psychiatric expert enjoying special allowances from the State Council of the People’s Republic of China, and one psychiatric expert honored with the title of “Double Creation Doctor” in Jiangsu Province, China, to improve the reliability of the study.
2. The interview outline was sent to the interviewees 1 week in advance to ensure that the questions could be fully answered, thereby improving the quality of the interview data.
3. To reduce human bias in the interviews, all interviews were conducted by one researcher, who was also one of the two data analysts.
4. The original data and analysis results were cross-validated using triangulation, and then reviewed by two other members of the research team.
5. The collated interview data were promptly fed back to the interviewees for them to confirm the accuracy of the results.
6. All members of the research team had good experience in qualitative research.

## 3 Results

A total of 14 parents of adolescents with emotional disorders caused by school bullying were interviewed in this study. All participants actively cooperated with the research procedures, and no participants withdrew during the interview process. All participants confirmed and agreed with the interview transcripts after data verification. One participant was interviewed twice due to the need for greater completeness and clarity of the interview content, resulting in a total of 15 interviews with a cumulative interview duration of 525 min.

During data analysis, the interview recordings were transcribed into 61,018 words of initial textual data. A total of 14 codes were established, including: unclear understanding of emotional disorders; inappropriate responses to patients’ emotional problems; anxiety; anger; regret; helplessness; self-blame; disappointment; pain, chest tightness, insomnia, poor appetite, general physical discomfort; learning from other parents; seeking help from mental health professionals; police officers entering schools for educational interventions; care and concern from teachers and peers; and online supervision/regulation.

A total of 462 coding instances were identified across the transcripts. The coded data were then aggregated and synthesized, resulting in the extraction of four overarching themes:

1. Insufficient understanding of emotional disorders caused by school bullying among adolescents;
2. Various negative psychological experiences;
3. Various forms of somatic discomfort;
4. A strong desire for support and assistance from multiple sectors of society.

A summary of the cognitive perceptions and psychosomatic experiences of the 14 participants regarding emotional disorders caused by school bullying is presented in Table 2.

**Table 2:** Interviewees’ cognition and psychosomatic experiences regarding emotional disorders caused by school bullying (*n* = 14).

Interviewee	Insufficient cognition of emotional disorders in adolescents caused by school bullying		Various negative psychological experiences						Various forms of somatic discomfort	Hope to obtain support and assistance from various sectors of society				
	Unclear concept of emotional disorders	Misconceptions about the disease	Anxiety	Anger	Regret	Helplessness	Self-blame	Disappointment	Pain, chest tightness, insomnia, poor appetite, and general physical discomfort	Hope to learn communication skills from parents who communicate well with their children	Hope psychologists can guide their children more and conduct more psychology education	Hope police can conduct more classes to prevent school bullying	Hope bullied children receive care from teachers and students after returning to school	Hope to strengthen network supervision
N1	+	+	+	+	−	+	+	+	+	−	+	−	+	+
N2	+	−	−	+	+	−	−	−	+	+	+	+	+	−
N3	+	+	−	−	+	+	+	−	−	−	+	+	+	−
N4	−	−	+	+	−	−	−	−	+	+	+	−	+	+
N5	+	+	−	+	+	+	+	+	−	−	+	+	+	+
N6	+	+	+	−	+	−	−	−	+	+	−	+	+	+
N7	+	+	−	+	−	+	−	+	−	+	+	−	+	−
N8	+	−	+	+	+	−	−	−	+	+	+	+	+	+
N9	−	−	+	−	+	+	+	−	+	−	+	−	−	−
N10	+	+	−	+	+	−	+	−	−	+	+	+	+	+
N11	+	+	+	−	+	−	+	−	+	−	+	−	+	−
N12	+	+	−	+	−	+	+	−	+	+	+	−	−	+
N13	+	−	+	−	−	+	−	−	−	−	+	+	+	−
N14	+	+	−	−	+	−	+	−	+	+	−	−	+	−
Total	12	9	7	8	9	7	8	3	9	8	12	7	12	7

“+” indicates “Yes,” “−” indicates “No,” and “Total” counts the number of “Yes” responses. “+” indicates presence; “−” indicates absence; “Total” counts the number of “presence”.

### 3.1 Theme 1: insufficient understanding of emotional disorders caused by school bullying

#### (1) Unclear concept

The interview results showed that only 2 out of 14 interviewees had heard about emotional disorders caused by school bullying before their children were hospitalized. The rest were completely unaware of the concept before hospitalization. After hospitalization, they began to have a vague understanding by analyzing their children’s experiences. For example, N1 said, “I had never heard of the term ‘emotional disorder.’ When the doctor told me, I asked several times to understand, but I still do not quite get it.” N14 said, “I never thought emotions could have disorders. It’s hard to understand, but combining it with my child’s behavior, I seem to understand a bit.”

#### (2) Misconceptions about the disease

Most interviewees had varying degrees of misconceptions about emotional disorders caused by school bullying. Some did not think their children’s state was a psychological problem, believing it was just an excuse to avoid school and did not require hospitalization, thinking they should ignore it. N7 said, “Who has not been in a fight at school? Who can be happy after a conflict? He did not like school before, and now he has more reasons. I think he does not need hospitalization; just beating him a few more times will be fine.” Some interviewees felt they lacked coping skills and placed all hopes on medical staff. N3 said, “We do not know how to treat illnesses or talk; we cannot persuade the child. Whether he can get better depends on the doctors and nurses.” Some believed that the child needed to retaliate equally to alleviate negative emotions. N6 stated, “As long as he fights back, it’s fine; otherwise, he’ll never find peace.” Others worsened their child’s emotional problems due to extreme or one-sided care methods. N5 said, “The expert said the child needs more companionship to increase his sense of security. I took leave to stay at home, even reducing housework to accompany him, serving his meals and clothes, basically not letting him out of my sight. But he found me annoying, locked me out of his room, and even shouted at me. I feel helpless (sigh).”

### 3.2 Theme 2: various negative psychological experiences

#### (1) Anxiety

Parents were worried about their child’s prognosis, academic performance, being discriminated against, and future employment difficulties. N4 said, “I do not know what will happen to my child—whether his emotions will improve, whether he can study, live, and work normally, whether he can have friends. If he does not get better, he will definitely be discriminated against when finding a partner.” N7 said, “My child used to have above-average grades. After getting sick, his grades dropped. He′s been hospitalized for half a month and has not read a book once. I’m very worried about his future grades; I do not even dare to think about the more distant future (rubs forehead with one hand).”

#### (2) Anger

Parents felt angry and could not understand why their child encountered such injustice, feeling they had nowhere to vent their anger. N8 expressed, “My child is so well-behaved but encountered bullies. I also want to fight back, but I know that’s not the best solution.” N5 said, “Every time I see my child isolating himself or even hurting himself, I get very angry. Why should my child suffer such pain?” (clenches fist).

#### (3) Regret

They regretted not paying enough attention to their child’s emotions and words before. N2 noted, “I used to care too little about my child and did not notice his changes. Now it’s useless to regret.” N10 said, “As parents, we have no experience. Both my wife and I are usually busy with work, neglecting the child’s emotions, thinking that providing for him is enough. Now it’s too late to say anything.”

#### (4) Helplessness

They did not know how to make up for their child or help him regain emotional stability. N12 said, “The child has not improved all along; I do not know what to do. Sometimes I just want to have a good cry (holds head with hand).” N3 said, “I have a low education level and do not know how to help my child come out of it; I do not even know how to persuade him.”

#### (5) Self-blame

They felt they had failed since their child was bullied. N9 said, “When my child was bullied, how helpless he must have felt. He probably really hoped someone would help him. What was I doing during those times?” (shakes head).

#### (6) Disappointment

They felt their child had poor psychological endurance and would achieve little in the future. N7 stated, “If you can fight back, fight; if you cannot, seek help. At 15 or 16, instead of finding ways to solve problems when encountering them, he chooses to escape. Won’t he be useless in the future?”

### 3.3 Theme 3: various forms of somatic discomfort

Participants most commonly reported somatic symptoms such as pain, chest tightness, insomnia, poor appetite, and nonspecific bodily discomfort that was difficult to clearly articulate. In severe cases, these symptoms impaired social functioning and led to a decline in quality of life. Such impairments were manifested as reluctance to perform household chores, difficulty maintaining concentration at work with reduced work efficiency, and even repeated help-seeking at general hospitals.

Participant N11 stated:

“Since discovering that my child became ill, I have not been able to eat or sleep well. I cannot concentrate at work, and I do not feel like doing housework at all. There’s no quality of life to speak of. Maybe when my child gets better, I will get better too.”

Participant N2 reported:

“When I first saw the injuries on my child’s arm, I felt like the sky had collapsed. I could not sleep, did not want to eat, had headaches and stomach pain, and felt uncomfortable all over. If my child can recover, maybe I can recover too—perhaps I may never recover.”

Participant N6 described:

“Since discovering my child’s illness, I have not been able to eat or sleep well. I feel uncomfortable all over my body, but I cannot clearly explain where. I do not feel like doing anything, though I can still force myself to go to work.”

Participant N12 stated:

“Physically, I often have headaches. I cannot think about my child—once I do, it feels like a heavy stone pressing on my chest, making it hard to breathe. I have no appetite, cannot sleep, feel physically weak, and often have headaches. Now, apart from taking care of my child, I do not feel like doing anything else.”

### 3.4 Theme 4: desire for support and help from society

#### (1) Hoping learn from parents who communicate effectively with their children

The interview results showed that out of 14 interviewees, 4 knew their child was bullied at school before the child became ill; the rest did not know. N6 said, “My child was bullied at school but never told me when he came home. I did not notice any emotional changes either; I thought he was just becoming introverted as he grew up. If I had known, I would have communicated with the teacher or the other parents in time. I really want to learn from parents who interact well with their children, see how they become friends with their kids, and have open communication.” N8 said, “When the child was bullied at school, her mother knew a bit but did not take it seriously, thinking it was just a minor conflict between students, and even advised her not to take it too seriously. Later, when the child refused to go to school and engaged in self-harm, her mother realized the seriousness and told me. I felt like an outsider before; neither the child nor her mother told me about the child’s situation. I never thought to ask proactively. I feel I did not play the role of a father well. I really hope an excellent father can guide me. I want to be my child’s support so she will not be afraid.”

#### (2) Hoping psychologists provide more guidance and psychological education

They hoped psychologists could care more about their children, conduct more popularization of psychological knowledge, teach parents and children methods to manage emotions, and help their children overcome psychological shadows and return to school smoothly. N11 said, “Different people have different communication abilities. When the child’s emotions are bad, listening to the expert’s Q&A with the child, after chatting, his emotions improve. When it’s our turn, it does not work. I hope the experts can care more about the child and talk to him more.” N9 said, “Seeing the child become like this, I feel upset and cannot help but lose my temper when things happen. Sometimes I’m not nice to doctors and nurses. I do not know how to help the child stabilize his emotions. I hope doctors and nurses can give us more lectures and teach us how to control our own emotions; this is very professional.”

#### (3) Hoping police officers conduct more classes to prevent school bullying

They hoped police officers could frequently hold classes to prevent school bullying, teach parents and children how to deal with it, and severely punish bullies. N10 said, “I hope police officers can often come to communities and schools to conduct legal education, deter those bad students and domineering parents, and teach ordinary students and parents how to protect themselves legally. For example, when I knew my child was bullied, my first thoughts were to find the teacher, the other parents, or even fight back, but I did not think to call the police first. It was my child’s aunt who called the police. Now, what I most want to see is the bully being punished by law.” N8 said, “Children have no legal awareness and do not realize they can solve most problems through legal means, let alone call the police immediately. Being threatened, they really do not dare to speak up. I hope frontline police officers can enter schools, give children courage, teach them to use legal weapons to protect themselves, and make those bad students afraid. This is more effective than us preaching legal knowledge to children.”

#### (4) Hoping the bullied child receives care from teachers and students upon returning to school

They hoped their children could regain confidence in school, complete their studies smoothly, teachers pay attention to feedback from students, proactively detect bullying behaviors, communicate with parents in time, and jointly help children stop bullying; they also hoped bullies would be punished by the school. N10 said, “Now the child’s emotions have significantly improved, and the next step is facing returning to school. I especially hope the school’s teachers and classmates can help my child more, care for him, let him feel the warmth from the school. I also hope the school punishes the bullies according to the regulations, which will help the child overcome his psychological shadow in the long term.” N13 said, “The child said that when he was bullied before, he also told the teacher. The teacher criticized those children on the spot, telling them not to bully my child again, but did not ask afterward. As a result, they continued to bully my child and threatened him not to tell the teacher. I just hope that after he goes back this time, teachers and classmates can pay more attention to my child. If he is bullied again, I hope teachers can intervene proactively.”

#### (5) Hoping to increase internet supervision

We aim to strengthen cyberspace regulation and establish a comprehensive multi-dimensional online regulatory system for minors featuring “the rule of law as the foundation, multi-stakeholder collaboration, and technological empowerment.” This system will clarify rights and responsibilities through institutional frameworks, pool synergistic efforts via multi-party coordination, enable children to fully embrace the conveniences brought by the development of the internet, and eradicate the breeding ground for cyberbullying. N1 said, “Now the internet is so developed; it’s normal for children to use mobile phones to go online. Originally, it’s a happy thing, but after a short time, she starts crying, saying someone is insinuating and scolding her again. It’s useless to tell her not to look. Compared to direct violent conflicts, this kind of cyberbullying is even more helpless.” N5 said, “The child is addicted to his phone; when awake, he basically cannot leave it. But he gets angry because of the content on the phone, often saying classmates are spreading rumors and attacking him. Each time, only the medical staff can persuade him to calm down; we cannot do anything.”

## 4 Discussion

### 4.1 Enhancing parents’ understanding of emotional disorders caused by school bullying

Participants demonstrated conceptual ambiguity and cognitive misconceptions regarding emotional disorders, a finding that is consistent with international research on families of bullying victims. Parents’ awareness of bullying is crucial for preventing its occurrence, early identification, and appropriate intervention (Deli et al., 2024). As the participants in this study were parents of adolescent patients and generally had lower levels of educational attainment, their understanding of disease-related knowledge was limited.

Most participants reported that their understanding of the disorder was vague and unclear, and expressed a need for explicit explanations regarding the definition of adolescent emotional disorders, common causes, clinical manifestations, commonly used treatment and care strategies, and prognosis. Providing families with a comprehensive and scientifically grounded understanding of the disorder is conducive to effective parental cooperation during the treatment process.

During the interviews, some participants believed that adolescents with emotional disorders did not have genuine psychological problems, but were merely attempting to avoid attending school, and therefore did not require treatment. Some even suggested that the problem could be resolved through corporal punishment. Such views reflect a lack of parental empathy, and these cognitions may be expressed in daily parent–child interactions, thereby becoming additional stressors affecting the child’s emotional state. Family violence, in particular, may serve as a trigger for extreme emotional reactions and behaviors in adolescents (Kim, 2021), which is highly detrimental to prognosis.

It is therefore essential to clarify that transient emotional distress is a common experience, whereas emotional disorders constitute a medical condition. Disorders characterized primarily by depression—especially when accompanied by suicidal behavior or non-suicidal self-injury (NSSI)—represent potentially life-threatening mental illnesses (Xin et al., 2022). Adolescents who experience bullying but fail to receive parental support face greater difficulties in emotion regulation, and may be more likely to adopt suicide or NSSI as maladaptive coping strategies (Emery et al., 2017). High levels of parental support can buffer the harmful relationship between bullying and negative emotions or self-harm, and can also significantly enhance adolescents’ social skills and facilitate social integration (Myklestad and Straiton, 2021; Cenușă and Turliuc, 2023).

Although the prevalence of suicide and NSSI behaviors tends to decline as adolescents reach adulthood, individuals with a history of such behaviors continue to face a relatively elevated risk of suicide (Zhang et al., 2022). Therefore, standardized and sustained treatment is particularly critical for long-term prognosis.

During the interviews, five participants reported that their children’s emotional problems should be resolved by retaliating through bullying, a stance that may be emotionally understandable but is legally and ethically unacceptable. Responding to violence with violence inevitably leads to mutual harm, whereas legal channels represent the most appropriate and effective means of resolution. In addition, seven participants placed all hope for their children’s emotional recovery entirely on healthcare professionals. This belief is also problematic. While treatment during the most acute phase of symptoms is indeed primarily led by medical professionals with family support playing a supplementary role, the maintenance of treatment effects relies predominantly on sustained family support rather than hospital-based care alone (Shang et al., 2022).

Furthermore, some participants mistakenly believed that the “accompaniment” recommended by professionals meant remaining physically present with the child at all times. In fact, the true meaning of accompaniment lies not in constant physical proximity, but in the ability to provide timely, positive responses whenever the child expresses a need (Xue et al., 2022b).

Notably, five participants reported that after a period of inpatient treatment and receiving psychoeducation from healthcare professionals, both they and their children had developed a new understanding of the disorder. These parents indicated that they were now able to jointly discuss with their children how to better cooperate with treatment and how to plan for future learning and daily life.

### 4.2 Guiding parents to rationally handle their negative emotional experiences and enhance psychological resilience

Psychological resilience refers to a process or capacity whereby an individual demonstrates better-than-expected psychological functioning or emotional outcomes after experiencing adversity or negative life events within a specific context (Troy et al., 2023). Parents’ own emotion regulation abilities play a critical role in shaping the development of children’s emotion regulation capacities (Paley and Hajal, 2022).

The interviews revealed that many participants were characterized by low income levels, largely influenced by educational attainment and occupational status, as well as poor marital relationship stability. These factors were found to be negatively correlated with psychological resilience, resulting in pronounced and persistent negative emotional experiences among parents following the onset of emotional disorders in their children.

Within traditional Chinese cultural values, children are often regarded as the hope of the family. Consequently, when a child exhibits abnormal emotional or behavioral manifestations, negative emotional reactions among parents and family members are almost inevitable. However, it should be clearly emphasized that the capacity to effectively manage one’s emotional experiences—whether positive or negative—is a core component of healthy psychological development (Paley and Hajal, 2022). Parents must first strive to maintain emotional stability, allowing their children to perceive a strong and reliable source of support behind them. As parents serve as primary role models, their ability to manage emotions effectively constitutes a powerful form of implicit teaching. Conversely, if parents become immersed in unprocessed negative emotions and are unable to extricate themselves, this may leave the child feeling more confused, overwhelmed, and emotionally constrained (Godleski et al., 2020).

Adolescents with emotional disorders are often highly sensitive and may exhibit cognitive distortions. They may misinterpret the negative emotions conveyed by parents’ psychological experiences—such as anxiety, anger, self-blame, regret, and helplessness—leading to negative internal attributions and further self-denial (Stavish and Lengua, 2023). Parents should therefore be particularly careful to avoid, whether intentionally or unintentionally, expressing disapproval or rejection toward the affected child through words or emotional attitudes, as such behaviors may precipitate irrational emotional outbursts and oppositional behaviors, exacerbate the child’s condition, and in extreme cases, jeopardize the child’s future or even result in loss of life (Manuele et al., 2023).

The family-level emotion coregulation framework proposed by Paley and Hajal likewise emphasizes that parents should avoid allowing their unprocessed emotions (e.g., disappointment, anger) to be transformed into disapproval or pressure directed at the child, thereby preventing the formation of negative interaction cycles that aggravate the child’s condition (Paley and Hajal, 2022).

If parents wish for their children to confront adversity with a positive mindset, they must first establish confidence themselves and learn to identify positive meanings within difficult situations. Parents should believe that their children will not remain permanently trapped in ruminations over adverse experiences, but can instead regard past experiences and illness as developmental challenges that strengthen psychological resilience. Emerging from such challenges, adolescents may ultimately find that broader possibilities and new opportunities lie ahead.

### 4.3 Multiple ways to improve parents’ physical feelings and restore their Normal social functioning and quality of life

Parents should be informed of the association between negative emotional experiences and somatic discomfort, and provided with explanations of the psychological foundations and physiological mechanisms underlying various somatic symptoms. This understanding can help parents accept their bodily experiences, thereby reducing indiscriminate or repetitive medical help-seeking behaviors. Parents should also be taught relaxation techniques, enabling them to alleviate somatic manifestations by improving maladaptive psychological states.

In addition, parents should be informed that maintaining normal work routines, daily activities, and social engagement can effectively divert attention and represent one of the most effective approaches for alleviating negative emotions and somatic discomfort. This encourages parents to actively re-engage in work, daily life, and social interactions. Guidance on appropriate physical exercise and participation in immersive experiential activities can further alleviate both psychological distress and somatic discomfort.

Moreover, parents should be encouraged to participate in collective and group-based activities, which have been shown to be more effective than individual activities in improving emotional states and somatic experiences. Collectively, these intervention strategies promote emotional socialization in parents, facilitate the development of healthy behavioral patterns, and ultimately contribute to the emotional socialization and emergence of positive behaviors in affected adolescents (Lebowitz et al., 2020; Hajal and Paley, 2020).

### 4.4 A comprehensive and diversified social support network

Most participants were employed as manual workers or farmers, had limited social networks, and were unable to rapidly identify effective solutions when confronted with difficulties. This limited access to resources was one of the key reasons why parents tended to place their hopes on external assistance. An individual’s sense of security is derived from strong support from family and society, as well as from the ability to effectively navigate interpersonal interactions in daily life (Yang et al., 2022).

In recent years, with the expansion of national informatization and the development of digital media, adolescents with emotional disorders caused by school bullying have increasingly entered the public eye. Online discussions have generated numerous suggestions, with five core recommendations emerging most prominently: (1) strict legal punishment of perpetrators; (2) public condemnation of bullying; (3) proactive school monitoring and firm disciplinary action against bullying, coupled with increased protection and care for victims; (4) full utilization of psychological professionals’ expertise to assist affected adolescents; and (5) guidance for parents to provide appropriate support and for adolescents to acquire coping skills to deal with bullying. These five recommendations were also repeatedly confirmed during interviews with parents of adolescents suffering from emotional disorders caused by school bullying.

Accordingly, it is recommended that the affected adolescent be placed at the center, and that existing resources—including law enforcement agencies, internet platforms, schools, healthcare institutions, families, and community volunteers—be fully integrated to enhance public concern and support for affected adolescents and their families.

(1) In the interview, some respondents expressed the desire to learn from parents who communicate effectively with their children. As one of the most important relational systems in adolescent development, the family constitutes a lifelong foundation for psychological health, and family functioning is closely associated with adolescents’ adaptation, self-concept formation, and capacity to cope with emotional distress (van Eickels et al., 2022). Parents hoped that experienced and compassionate individuals could connect with them via one-to-one communication or through WeChat and QQ groups, facilitating shared discussions about parent–child communication strategies. Such peer support could promote parents’ rapid growth and help alleviate existing family stress (Yang et al., 2022).

(2) Professional psychological support and school reintegration assistance. The vast majority of parents expressed a strong desire for greater involvement from mental health professionals, including increased dissemination of psychological knowledge and assistance with successful school reintegration. It is recommended that mental health institutions and psychological associations regularly offer community-, hospital-, and school-based psychoeducational programs, focusing on emotion regulation strategies and parent–child communication skills. These initiatives aim to help primary family members form an ideal family functioning model, cultivate proactive emotional management awareness, and clarify role responsibilities. Parents and children should be encouraged to become each other’s primary recipients of shared emotions, thereby reducing communication gaps and supporting adolescents’ recovery from psychological trauma. Child and adolescent psychiatric wards are encouraged to establish school reintegration support programs, enabling parents to understand adolescent psychological characteristics, learn to listen to children in distress, recognize common psychological problems, and reflect on the underlying causes of reintegration difficulties. Parents should be guided on how to discuss returning to school with their children, and how healthcare professionals, parents, and adolescents can collaborate by adjusting sleep routines, jointly formulating and implementing electronic device use guidelines, gradually reducing excessive screen time, and understanding administrative procedures related to school reintegration. Mental health institutions may also establish specialized outpatient services for school reintegration, providing staged psychological counseling to support adolescents’ gradual return to campus.

(3) Law enforcement involvement and preventive education. Some parents suggested that law enforcement officers should provide more educational lectures on the prevention and management of school bullying and ensure strict punishment of perpetrators. It is recommended that local police stations systematically conduct training programs within their jurisdictions—including communities, schools, enterprises, and institutions—to help parents and teachers identify early signs of bullying and equip adolescents with effective coping strategies, thereby enabling bullying incidents to be terminated promptly. The Guiding Opinions on the Prevention and Control of Bullying and Violence among Primary and Secondary School Students, jointly issued in November 2016 by nine Chinese government departments (Ministry of Education, et al., 2016), clearly stipulates that perpetrators should be punished by law once bullying is confirmed, a response that also aligns with victims’ most fundamental expectations and enhances public trust in law enforcement.

(4) School-based care, disciplinary systems, and tiered support mechanisms. Some parents hoped that bullied children would receive care and support from teachers and peers upon returning to school, and that perpetrators would face appropriate disciplinary measures. A strong sense of school belonging and well-being can significantly reduce bullying-related negative emotional experiences, lower the likelihood of extreme behaviors, improve peer relationships, and increase opportunities for mutual assistance (Myklestad and Straiton, 2021; Cenușă and Turliuc, 2023; Zhang et al., 2022; Shang et al., 2022; Xue et al., 2022b; Troy et al., 2023; Paley and Hajal, 2022; Godleski et al., 2020; Stavish and Lengua, 2023; Manuele et al., 2023; Paley and Hajal, 2022; Lebowitz et al., 2020; Hajal and Paley, 2020; Yang et al., 2022; van Eickels et al., 2022; Ministry of Education, et al., 2016; Wang and Chen, 2023). School-based interventions have been shown to effectively reduce bullying incidence (Díaz-Caneja et al., 2021). Schools are therefore advised to establish regular psychological counseling services, provide 24-h consultation access, conduct periodic mental health screenings, and implement targeted interviews and interventions for students at risk of emotional disorders. Schools should emphasize root-cause identification and comprehensive intervention, establish collaborative agreements with healthcare institutions, and create referral mechanisms and green channels. Meta-analytic evidence from Gaffney et al. confirms that the most effective anti-bullying programs involve whole-school participation and multi-component collaboration (Gaffney et al., 2021). Schools are encouraged to implement a three-tiered support system:

- Primary-level support: peer-pairing and mutual assistance. Adolescents are more willing to communicate with trusted peers and more receptive to peer advice (Hale et al., 2023). Being perceived as “different” is a key predictor of peer victimization (Manuele et al., 2023). Facilitating peer integration, fostering empathy, and encouraging classmates to intervene during bullying incidents are effective strategies to reduce bullying (Juvonen and Graham, 2014).
- Secondary-level support: teacher-led individual management. For students with prior bullying experiences or negative emotional indicators identified through screenings, schools should adopt a one-to-one management model, with teachers assigned to specific student groups and conducting regular follow-up interviews. Teachers should collaborate with parents to enhance adolescents’ social skills and reduce interpersonal conflicts (Juvonen and Graham, 2014).
- Tertiary-level support: school administrative intervention. Bullying is associated with family demographic characteristics, and adolescents from socially disadvantaged backgrounds face higher risks. Schools should develop policies that prioritize early identification and intervention for these students to reduce adverse outcomes (Wang et al., 2021). Additionally, schools should install principal’s mailboxes in both high- and low-traffic areas, publicize their function, and provide students with confidential channels for help-seeking.

This tiered framework offers comprehensive protection, contributes to bullying prevention, and facilitates recovery among adolescents with emotional disorders. Furthermore, orientation sessions on school disciplinary regulations serve as deterrence, education, and enforcement foundations. Once bullying is confirmed, prompt disciplinary action should be taken to support moral development and foster a positive campus climate. The school regularly conducts special campaigns against “campus bullying” every half year or annually. These campaigns evaluate the implementation of the school’s previous anti-"campus bullying” measures, assess the actual occurrence of “campus bullying,” and, as needed, review the handling procedures for such incidents. The aim is to identify gaps in the work, make timely improvements, and dynamically adjust the school’s anti-"campus bullying” measures to keep them up-to-date and effective. Ultimately, the goal is to achieve a gradual annual decrease in the incidence rate of “campus bullying.”

(5) In interviews, some parents expressed hope for establishing a comprehensive supervision system for minors’ online activities, so that cyberbullying can find no place to hide. Digital environments allow adolescents to transcend traditional physical boundaries, enabling online social interaction while also exposing them to conflict and harm (Lee et al., 2017). The internet is not beyond the reach of law. In cases of cyberbullying, parents’ active participation and joint coping, alongside legal action, constitute crucial psychological support for adolescents and play a key role in mitigating harm (Xue et al., 2022a). Active and passive intervention by platform administrators is central to reducing cyberbullying, including promoting civil online discourse, rigorously verifying images and videos, rejecting misinformation and personal attacks, combating rumor dissemination, and fostering a healthy, positive, and orderly online environment.

## 5 Conclusion

In summary, this study conducted in-depth interviews with 14 parents of adolescents who developed emotional disorders as a result of school bullying. The findings indicate that parents demonstrated insufficient understanding of emotional disorders caused by school bullying. After becoming aware of their children’s diagnosis, parents experienced intense and persistent negative psychological reactions and somatic discomfort, which in some cases led to impaired social functioning and reduced quality of life. Participants consistently emphasized that terminating or reducing school bullying and mitigating its long-term consequences requires broad support and assistance from multiple sectors of society.

Therefore, it is essential to systematically enhance parents’ knowledge of emotional disorders caused by school bullying, enabling them to adopt a scientifically informed and appropriate attitude toward the condition. At the same time, greater attention must be given to addressing and actively alleviating parents’ negative emotional experiences and somatic discomfort through targeted interventions. Efforts should be made to establish and refine a comprehensive prevention, management, and support system, jointly involving law enforcement agencies, internet platforms, schools, healthcare institutions, families, and community volunteers. Ultimately, these measures may facilitate the early alleviation of psychological distress in both adolescents affected by bullying-related emotional disorders and their parents, support the restoration of emotional stability, and promote a full return to normal social and familial roles.

### 5.1 Theoretical and practical implications

This study contributes meaningfully at both the theoretical and practical levels. Theoretically, it extends the focus of school bullying research from bullied adolescents alone to the broader family system, offering an initial depiction of the complex patterns of illness perceptions and psychosomatic experiences among parents as secondary stakeholders. Furthermore, the study exploratorily applies the illness perception model to emotional disorders triggered by external social trauma (bullying), providing a potential theoretical framework for understanding how parents construct cognitive meanings and coping responses during such crises, and offering preliminary insights into the model’s applicability across different cultural contexts.

### 5.2 Limitations

Despite the valuable insights provided, this study has several limitations.

First, cultural and regional specificity: all participants were recruited from a single mental health center in eastern China, and their experiences may have been influenced by local educational resources, healthcare accessibility, and regional cultural characteristics. Caution is therefore warranted when generalizing the findings to other cultural contexts, healthcare systems, or rural settings.

Second, methodological limitations: as a qualitative study, the primary aim was to explore and gain in-depth understanding rather than to establish quantitative relationships. Future research could adopt mixed-method approaches, such as conducting large-scale surveys based on qualitative findings using instruments like the Illness Perception Questionnaire–Revised (IPQ-R) and the Patient Health Questionnaire-15 (PHQ-15), to quantify the associations and prevalence of parental illness perceptions and psychosomatic symptoms.

Finally, recall bias: interview data relied on parents’ retrospective accounts of past experiences, which may have been influenced by current emotional states, social desirability, or memory distortion over time. Future longitudinal studies could conduct follow-up interviews beginning at the time of the child’s diagnosis, allowing for more real-time capture of dynamic changes in parents’ cognition and experiences.

## Data Availability

The original contributions presented in the study are included in the article/supplementary material, further inquiries can be directed to the corresponding authors.
